# Emerging Approaches in Breast Cancer: From Molecular Mechanisms to Diagnosis and Therapeutic Strategies

**DOI:** 10.32604/or.2026.081924

**Published:** 2026-06-16

**Authors:** Raquel Sanchez-Baltasar, Nerea Castañeda-Fernández, Jorge Olivares-Arancibia, Carlos Torres-Villar, Julio Plaza-Diaz, Lourdes Herrera-Quintana

**Affiliations:** 1 Laboratory of Cellular and Molecular Gerontology, Precision Nutrition and Aging, Madrid Institute for Advanced Studies—IMDEA Nutrition, CEI UAM+CSIC, Madrid, Spain; 2 AFySE Group, Research in Physical Activity and School Health, School of Physical Education, Faculty of Education, Universidad de Las Américas, Santiago, Chile; 3 Programa de Doctorado en Ciencias Morfológicas, Facultad de Medicina, Universidad de La Frontera, Temuco, Chile; 4 Departamento de Ciencias Morfológicas, Facultad de Ciencias, Universidad San Sebastián, Puerto Montt, Chile; 5 Departament de Bioquímica i Biotecnologia, ANUT-DSM (Alimentaciò, Nutrició Desenvolupament i Salut Mental), Universitat Rovira i Virgili, Reus, Spain; 6 Institut de Recerca Biomèdica Catalunya Sud, Hospital Universitari Sant Joan de Reus, Reus, Spain; 7 Biomedical Research Networking Center for Physiopathology of Obesity and Nutrition (CIBERObn), Institute of Health Carlos III, Madrid, Spain; 8 School of Health Sciences, Universidad Internacional de La Rioja, Logroño, Spain

**Keywords:** Breast cancer (BC), digital pathology, liquid biopsy/ctDNA, antibody–drug conjugates, precision oncology, artificial intelligence (AI)-enabled diagnostics

## Abstract

Breast cancer (BC) is the most frequently diagnosed malignancy in women worldwide and remains one of the leading causes of cancer-related mortality, with substantial international disparities in incidence, stage at diagnosis, access to treatment, and survival. In recent years, BC management has evolved rapidly through advances in molecular characterization, imaging, pathology, targeted therapies, immunotherapy, and survivorship care. Nevertheless, important gaps persist in early and accurate detection, biomarker standardization, equitable access to care, and patient-specific treatment selection. These advances require timely, evidence-based, and context-specific clinical frameworks to support appropriate implementation, and to avoid the use of costly interventions with limited or uncertain clinical benefit and/or low-impact therapies with no clear therapeutic value. This review aims to provide a comprehensive overview of the molecular pathogenesis of BC and to synthesize current advances across diagnosis, therapeutic strategies, and clinical implementation. We examine liquid biopsy (ctDNA/circulating tumor cells) for early detection and minimal residual disease monitoring, alongside the transformative role of multi-omic molecular profiling and artificial intelligence (AI). Therapeutic section covers different options of treatments, from standard therapies to specific targeted therapies (e.g., human epidermal growth factor receptor (HER2-), DNA damage repair–, and cell-cycle–directed agents). We also address resistance and tumor heterogeneity, implementation and equity barriers, and survivorship needs (toxicity, quality of life, cardio-oncology). Collectively, emerging technologies and integrated platforms offer the potential measurable improvements in diagnostic precision, treatment efficacy, and patient outcomes, provided that validation, harmonization, and equitable adoption progress at a similar pace.

## 1 Introduction

Breast cancer (BC) is a highly heterogeneous disease driven by a complex interplay of genetic predisposition and non-genetic factors, including lifestyle, environmental exposures, and hormonal factors [1]. BC remains a major global health challenge nowadays, with an estimated 2.3 million new cases and 670,000 deaths in 2022, with incidence continuing to rise in many countries [2]. Public health projections suggest a substantial increase by 2050, with the annual number of cases expected to exceed 6 million cases [3]. This high incidence places BC at the forefront of oncological diseases worldwide and underscores its substantial impact on healthcare systems, patients, families, and societies [1]. However, global patterns of mortality are more complex than a simple association with socioeconomic status, and mortality-to-incidence ratios vary across regions without strictly mirroring income level differences. For instance, some regions with differing socioeconomic profiles may exhibit comparable mortality-to-incidence ratios, highlighting the need for cautious interpretation of epidemiological disparities. Moreover, factors such as differences in screening practices, including potential overdiagnosis in countries with high Human Development Index, as well as variability in healthcare access and treatment quality, contribute to these patterns. Therefore, while disparities in early detection and treatment access remain important considerations, epidemiological data should be interpreted within a broad context [2,4]. Limited screening coverage and diagnostic capacity are associated with a higher proportion of late-stage disease, which is a well-established predictor of low survival, underscoring the importance of early detection and robust diagnostic pathways in improving outcomes [5].

The etiology of BC has multiple risk factors that contribute to its global burden, with metabolic and behavioral factors (e.g., alcohol consumption, smoking, dietary patterns, and physical inactivity) representing a major proportion of attributable risk [6]. Additional determinants include age, race and ethnicity, socioeconomic status, genetic susceptibility, and hormonal and reproductive variables [7]. Thus, both the prevention and detection of BC are still challenging, particularly because inequitable access to screening and diagnostic services remains one of the most significant barriers. Screening mammography, which improves the detection of early-stage cancers and can reduce mortality, remains underutilized in many regions [8]. In addition, BC treatment faces important challenges because the intrinsic biological diversity of breast tumors necessitates comprehensive management strategies including surgery, radiotherapy, systemic chemotherapy, targeted therapies, and precision oncology approaches [9]. Subtypes such as triple-negative breast cancer (TNBC) and human epidermal growth factor receptor 2 (HER2, also known as ERBB2)-enriched tumors often require aggressive and tailored therapeutic strategies that can be both costly and resource-intensive. Even in high-resource settings, the optimal integration of targeted therapies, immunotherapies, and novel agents into clinical practice continues to evolve, with ongoing debate regarding cost-effectiveness and long-term survival benefit [10]. At the same time, emerging technologies, including artificial intelligence (AI)-based diagnostic tools and deep learning models for imaging analysis, offer promising improvements in detection accuracy. However, the translation of these innovations into routine clinical practice remains constrained by issues related to model generalizability, interpretability, integration into clinical workflows, and validation across diverse populations [11].

Taken together, the complex interactions among risk factor prevalence, healthcare infrastructure, surveillance capacity, and the effectiveness of cancer control strategies highlight the need for innovative approaches, a better understanding of the underlying mechanisms of BC, and the integration of new diagnostic and treatment alternatives [12]. Accordingly, the aim of this review is to provide an overview of emerging diagnostic modalities, therapeutic strategies, and integrated care models, while summarizing current evidence and future perspectives in BC management.

## 2 Molecular Pathogenesis and Subtypes of BC

### 2.1 Tumor Classification and Clinical Implications

BC is a highly heterogeneous disease characterized by diverse morphological and biological features, clinical behaviors, and treatment responses, largely driven by underlying genetic and epigenetic variation.

Over time, BC classification has undergone substantial refinement as a consequence of progressive insights into tumor biology and advances in high-throughput molecular profiling technologies. Currently, breast tumors are stratified using three complementary, yet not entirely overlapping, approaches: histological classification, immunohistochemical-based surrogate subtypes, and intrinsic molecular subtypes derived from gene expression profiling [13]. Although histologic classification remains the basis of routine BC diagnosis, complementary stratification using immunohistochemical (IHC) surrogate subtypes and intrinsic molecular subtypes provides additional biological and clinical insight. Histologic classification describes tumor architecture and cytology, whereas IHC surrogate subtypes, based on estrogen receptor (ER), progesterone receptor (PR), and HER2, and Ki-67 expression, approximate molecular subtypes and guide treatment decisions in everyday practice. Intrinsic molecular subtypes, defined by gene expression profiling such as PAM50, capture deeper tumor heterogeneity and offer prognostic and predictive value beyond morphology and IHC. While molecular assays are increasingly integrated into selected clinical settings, IHC surrogate subtyping remains the most widely used approach in routine practice due to its accessibility and cost-effectiveness, with molecular profiling serving as a complementary tool for refining prognosis and informing personalized therapy [14,15].

#### 2.1.1 Histological Classification

Historically, breast tumors have been classified as ductal or lobular carcinomas according to their site of origin. Lesions that invade surrounding tissue are designated as invasive carcinomas, whereas those confined to the site of origin are classified as ductal carcinoma *in situ* or lobular carcinoma *in situ*. Among *in situ* carcinomas, ductal carcinoma is the most prevalent and can be further subclassified into five histological patterns: comedo, cribriform, micropapillary, papillary, and solid [16,17]. Invasive carcinomas are further classified into seven histological subtypes: invasive ductal carcinoma, invasive lobular carcinoma, ductolobular, mucinous, tubular, medullary, and papillary carcinomas. Invasive ductal carcinoma is the most common subtype and is graded as well-differentiated (grade 1), moderately differentiated (grade 2), or poorly differentiated (grade 3), reflecting increasing degrees of cellular atypia and biological aggressiveness [18,19]. These histological features, particularly tumor type and grade, are routinely reported in clinical pathology and play a direct role in prognostic assessment and therapeutic decision-making [20].

#### 2.1.2 Immunohistochemical Classification

Because histopathological classification alone provides limited prognostic information, immunohistochemical biomarkers, such as ER, PR, and HER2, have been incorporated into routine tumor stratification. Based on the expression patterns of these markers, breast tumors are commonly categorized into four principal surrogate subtypes: luminal A, luminal B, HER2-enriched, and TNBC. This classification has demonstrated robust prognostic relevance in invasive breast carcinomas, although its utility remains more limited in carcinoma *in situ* [21,22]. Importantly, these IHC-based surrogate subtypes approximate but do not fully recapitulate intrinsic molecular subtypes defined by gene expression profiling, and discrepancies between both approaches have been reported [23,24].

Among these subtypes, luminal A tumors are the most prevalent and are typically associated with lower histological grade, more favorable prognosis, and higher 4-year survival rates. In contrast, luminal B tumors exhibit higher proliferative activity, greater histological grade, and more aggressive clinical behavior, resulting in poorer survival outcomes relative to luminal A tumors [25,26]. By contrast, HER2-enriched tumors generally display intermediate-to-high histological grade. Importantly, the introduction of HER2-targeted therapies, particularly monoclonal antibodies, has markedly improved clinical outcomes and significantly increased 4-year survival in this subgroup [27]. Finally, TNBC is the most aggressive subtype and is associated with the poorest survival outcomes. These tumors predominantly affect younger women and are characterized by the absence of hormone receptor (HR) and HER2 expression, rendering endocrine and HER2-targeted therapies ineffective and thereby substantially limiting treatment options [28,29]. In routine clinical practice, ER, PR, HER2, and proliferation markers such as Ki-67 are systematically evaluated and directly guide therapeutic decisions, including the use of endocrine therapy, anti-HER2 agents, and chemotherapy [20,30].

#### 2.1.3 Molecular Classification

To further refine prognostic stratification and guide therapeutic decision-making, advances in gene expression profiling have enabled molecular classification systems based on transcriptional signatures. Pioneering studies identified distinct molecular portraits of BC and proposed classification into five intrinsic subtypes: luminal A, luminal B, HER2-enriched, basal-like, and normal-like [31]. These findings subsequently led to the establishment of the PAM50 gene expression signature, which includes genes associated with HR signaling, cellular proliferation, and basal/myoepithelial differentiation [32]. Clinically validated assays based on PAM50 are currently used in selected clinical settings to estimate recurrence risk and support decisions regarding adjuvant therapy, particularly in HR–positive BC [33]. Subsequent refinements to molecular taxonomy also identified claudin-low tumors as a biologically relevant subgroup. Importantly, intrinsic molecular subtyping has demonstrated significant prognostic and predictive value and is increasingly integrated into clinical decision-making algorithms for BC management, although its implementation remains complementary to standard pathology rather than replacing it [34,35].

### 2.2 Key Molecular Pathways in Initiation and Progression of BC

BC arises through alterations in tumor suppressor genes and oncogenes, driven in part by transcription factors such as NF-κB, which regulates gene networks involved in inflammation, cell proliferation, metastasis, and apoptosis, thereby promoting tumor development [36]. Likewise, BC progression results from tightly interconnected signaling networks in which receptor tyrosine kinases (RTKs), including HER2, EGFR, HER3, FGFR1, and MET, act as major upstream regulators. Upon activation, these receptors initiate key downstream cascades, including PI3K/AKT/mTOR and RAS/MAPK, while interacting with additional pathways such as non-canonical Wnt/planar cell polarity and Notch/JAK/STAT. Together, these pathways integrated signals from the tumor microenvironment (TME), with intracellular programs that regulate proliferation, survival, metabolic reprogramming, invasion, epithelial–mesenchymal transition (EMT), and stemness, thereby coordinating metastatic dissemination and overall disease progression [37,38].

#### 2.2.1 Receptor Tyrosine Kinases

RTKs, including HER2, EGFR, and IGF-1R, are frequently overexpressed or amplified in breast tumors, leading to activation of downstream PI3K/AKT and RAS/MAPK signaling. As previously outlined, BC molecular subtypes are defined by distinct transcriptional programs, which are closely linked to differential activation of these signaling pathways. Thus, the alterations described below should be interpreted within the context of subtype-specific biology.

The PI3K/AKT/mTOR pathway is central to BC initiation and progression by regulating cell proliferation, survival, metabolism, and therapy resistance. PI3K catalyzes the conversion of PIP2 to PIP3, recruiting AKT to the membrane where it is phosphorylated and activated. Activated AKT promotes proliferation, inhibits apoptosis, and influences metabolism and protein synthesis via mTOR complexes. Alterations in components such as PIK3CA, PTEN, and AKT1 are common across molecular subtypes of BC, with PIK3CA mutations detected in up to 45% of tumors and associated with distinct prognostic impacts dependent on mutation site and subtype [39,40]. Functional activation of AKT and downstream mTOR promotes oncogenic growth and may influence response to targeted therapies such as CDK4/6 inhibitors in metastatic settings [41,42]. Early activation of this pathway in mammary carcinogenesis may also promote EMT and cancer cell dissemination [43].

Concurrently, RTK activation recruits adaptor proteins such as GRB2 and SOS, which facilitate RAS activation. Active RAS initiates the RAF-MEK-ERK kinase cascade, promoting transcriptional programs that drive cell growth and proliferation. Although RAS mutations are relatively infrequent in BC, RAS/MAPK signaling is activated in a substantial proportion of tumors through upstream RTK overexpression, contributing to aggressive phenotypes and reduced sensitivity to targeted therapies [44,45]. Alterations in HER2 and PI3K/AKT signaling are clinically relevant. HER2 amplification predicts response to trastuzumab, pertuzumab, and antibody-drug conjugates, while PIK3CA mutations guide the use of PI3K inhibitors (such as alpelisib) in HR-positive BC [46,47]. RTK-related alterations are also being evaluated as liquid biopsy biomarkers through circulating tumor DNA (ctDNA) assays for early detection and therapy monitoring [48].

#### 2.2.2 Non-Canonical Wnt Signaling

Another pathway implicated in BC pathogenesis is non-canonical Wnt signaling through planar cell polarity components such as VANGL1 and VANGL2, which promote tumor progression by enhancing collective cell migration and metastasis independently of primary tumor growth. Suppression of VANGL2 reduces cell motility and distant metastases, supporting a role for Wnt/planar cell polarity signaling in invasive behavior. This collective migration may complement canonical EMT-dependent invasion and integrates with cytoskeletal regulators such as RhoA [41,49]. Emerging evidence suggests that non-canonical Wnt components may serve as biomarkers for aggressive or metastatic BC. Preclinical studies are exploring these molecules as potential therapeutic targets, and integration with EMT-related signatures or circulating tumor cell analyses could inform patient stratification and therapy monitoring [41,50].

#### 2.2.3 Notch Pathway

The Notch signaling pathway also contributes to cell fate determination and tumor progression. In BC, Notch3 functions as a regulator of metastasis, partly by transcriptionally activating STAT5A, which inhibits mobility, invasion, and EMT *in vitro*; accordingly, its expression has been linked to better prognosis [51,52]. JAK/STAT signaling, particularly through STAT proteins, links extracellular cytokine signals to transcriptional programs that regulate growth and immune interactions. Dysregulated STAT activity is frequently observed in BC and correlates with aggressive phenotypes. Recent studies demonstrate Notch signaling is not only involved in basic tumor biology but also has implications for prognosis and potential therapy. Specifically, Notch3 regulation of STAT5A has been linked to metastatic suppression and better outcomes in BC patients, suggesting its potential as a prognostic biomarker and therapeutic target [49]. Moreover, modulation of the Notch pathway is being explored in preclinical and early clinical contexts, particularly in aggressive subtypes such as TNBC [53], and its interaction with tumor immunity and angiogenesis indicates new therapeutic approaches [54].

#### 2.2.4 Hormone Receptors

HRs are critically involved in BC pathogenesis. ER and PR pathways frequently intersect with growth factor signaling. ER activation enhances the expression of growth factor receptors and downstream effectors, enabling both genomic and non-genomic signaling that converges on PI3K/AKT and MAPK pathways. HER2 can also phosphorylate ER at specific residues, enabling ligand-independent transcriptional activity and facilitating endocrine therapy resistance. Tumors co-expressing HR and HER family members exhibit complex feedback loops that sustain cell proliferation and survival [55]. Recent clinical and translational evidence indicates that alterations in HR signaling, particularly through ESR1 mutations and pathway crosstalk with PI3K/AKT/mTOR and HER family signaling, have direct implications for endocrine therapy resistance and guide emerging targeted strategies. ESR1 mutations leading to ligand-independent ER activity are associated with resistance to aromatase inhibitors and other endocrine therapies [56]. Furthermore, the interplay between ER signaling and downstream pathways (e.g., PI3K/AKT and MAPK) underpins the rationale for combining endocrine therapy with PI3K inhibitors (e.g., alpelisib) or CDK4/6 inhibitors, strategies that have shown improved progression-free survival in HR-positive/HER2-negative metastatic BC [57,58].

#### 2.2.5 Cell-Cycle Regulation and DNA Damage Response

The integrity of cell-cycle checkpoints and DNA damage response pathways is essential for genomic stability. Cyclin D1 amplification and CDK4/6 activation promotes RB phosphorylation, permitting G1-to-S phase progression. Alterations in TP53, which are prevalent in basal-like and TNBC subtypes, impair apoptotic responses and DNA repair, thereby promoting genomic instability. Mutations in DNA damage response genes such as BRCA1/2, CHEK2, ATM, and ATR are associated with defective DNA repair and elevated mutation burden, contributing to tumor initiation and progression [59,60]. Defects in cell cycle regulation and DNA damage response have direct therapeutic implications. CDK4/6 inhibitors, including palbociclib, ribociclib, and abemaciclib, target cyclin D1–CDK4/6–RB signaling and are standard therapy for HR-positive advanced BC, improving progression-free and overall survival [61]. TP53 alterations are linked to therapy resistance and may inform patient stratification in clinical trials [62]. Homologous recombination deficiency, due to mutations in BRCA1/2 or related genes, predicts sensitivity to PARP inhibitors and platinum-based chemotherapy, and companion diagnostic assays are increasingly used to select patients for these targeted therapies [63,64,65].

#### 2.2.6 Epithelial-to-Mesenchymal Transition

EMT is a highly regulated developmental program that is aberrantly activated in carcinomas, allowing epithelial cells to acquire mesenchymal characteristics, including enhanced motility and invasiveness. Although EMT is well characterized at the molecular level, its assessment in routine pathology remains limited. Immunohistochemical evaluation of EMT-related markers (such as E-cadherin, N-cadherin, and vimentin) is not currently standardized in clinical guidelines and is mainly confined to research settings or used as an adjunct in selected diagnostic contexts [66]. Loss of E-cadherin and gain of N-cadherin and vimentin are hallmarks of EMT. Transcription factors such as SNAIL, SLUG, TWIST, and ZEB repress adhesion genes and activate genes involved in motility and invasion. EMT is driven by multiple signaling inputs, including TGF-β, Wnt/β-catenin, Notch, and RTKs, and is associated with stemness, resistance to apoptosis, and metastatic dissemination. Integrin-mediated adhesion to the extracellular matrix activates focal adhesion kinase and cooperates with PI3K and MAPK signaling to enhance motility and invasive capacity [67,68,69]. Despite its biological relevance, the direct clinical utility of EMT as a biomarker remains under investigation, and it is not currently used as an individual parameter for therapeutic decision-making [66].

### 2.3 Genetic and Epigenetic Alterations

#### 2.3.1 BRCA1 and BRCA2

BRCA1 and BRCA2 are critical tumor suppressor genes involved in homologous recombination–mediated repair of DNA double-strand breaks. Germline pathogenic variants in these genes confer a markedly increased risk of BC and account for a substantial proportion of hereditary BC cases [70].

BRCA1-associated tumors are frequently high-grade, TNBC, and genomically unstable [71]. Functional loss of BRCA1 or BRCA2 leads to homologous recombination deficiency, promoting chromosomal instability and accumulation of secondary oncogenic alterations [70]. In addition to germline mutations, epigenetic silencing of BRCA1 through promoter hypermethylation has been identified in sporadic BC and is associated with reduced gene expression and HRD-like phenotypes [72,73]. This BRCA deficiency has important therapeutic implications, particularly in relation to platinum-based chemotherapy and PARP inhibitors.

#### 2.3.2 TP53

TP53 is the most frequently mutated tumor suppressor gene in human cancer. In BC, somatic TP53 mutations occur in approximately 30–35% of cases overall, with significantly higher frequencies in TNBC and HER2-enriched subtypes [59]. Analyses have demonstrated that these mutations follow sub-type specific patterns, with missense mutations predominating and often conferring gain-of-function oncogenic properties [74]. The Cancer Genome Atlas molecular profiling study confirmed that TP53 mutations are enriched in basal-like tumors and are associated with genomic instability and poor prognosis [59]. TP53 dysfunction compromises cell-cycle arrest, apoptosis, and DNA repair pathways, thereby facilitating tumor progression and therapeutic resistance [75].

#### 2.3.3 PI3K/AKT Pathway Mutations

As noted above, activation of the PI3K/AKT signaling pathway is among the most common oncogenic events in BC. PIK3CA, which encodes the catalytic subunit p110α of PI3K, is mutated in approximately 30–45% of BC cases, particularly in HR-positive tumors [76,77]. The most frequent hotspot mutations occur in the helical (E542K, E545K) and kinase (H1047R) domains [77]. Large-scale sequencing studies have shown that PIK3CA mutations are early clonal events and are associated primarily with luminal subtypes [59,76]. Functional studies have demonstrated that these mutations promote oncogenic transformation and aberrant AKT activation [77]. AKT1 mutations occur in a smaller subset of BC (approximately 2–4%) and result in constitutive membrane localization and pathway activation [78]. Loss of PTEN, a negative regulator of PI3K signaling, occurs via mutation, deletion, or epigenetic silencing and is particularly frequent in TNBC. PTEN deficiency leads to unchecked AKT activation and enhanced cell survival [79].

#### 2.3.4 DNA Methylation

Aberrant DNA methylation is also one of the hallmarks of carcinogenesis. Genome-wide methylation profiling has revealed subtype-specific methylation signatures that cooperate with genetic alterations in BC [80]. BRCA1 promoter hypermethylation is observed in 9–24% of sporadic BCs and is associated with reduced protein expression. Importantly, BRCA1 methylated tumors show a highly similar phenotype to that of germline-mutant tumors at the molecular level [81]. RASSF1A, CDH1, and other tumor suppressor genes are frequently hypermethylated in BC, contributing to the silencing of growth-regulatory pathways [82]. However, despite its biological significance, DNA methylation analysis is not yet routinely implemented in standard BC diagnosis and is mainly used in research or exploratory biomarker studies, although it holds promise for future clinical applications [83].

#### 2.3.5 Histone Methylation, Acetylation, and Deacetylation

Epigenetic dysregulation in BC is not limited to DNA methylation; it also includes extensive alterations in histone modification patterns and chromatin remodeling machinery, leading to the reprogramming of gene expression and modulation of tumor phenotype [84]. One of the most extensively studied histone modifiers in BC is Enhancer of Zeste Homolog 2 (EZH2), the catalytic subunit of the Polycomb Repressive Complex 2, which mediates trimethylation of histone H3 at lysine 27. EZH2 overexpression has been associated with aggressive tumor behavior, basal-like phenotype, high proliferative activity, and poor clinical outcome [85]. Functional studies have demonstrated that overexpression of EZH2 promotes oncogenic transformation and tumor progression through transcriptional silencing of tumor suppressor genes and modulation of cell-cycle regulators [84,86]. Notably, EZH2 expression in normal tissue has also been proposed as a prognostic indicator of premalignant breast lesions [87]. Importantly, EZH2 overexpression is particularly enriched in HER2-enriched, TNBC and basal-like and BC, where it contributes to stem-cell–like properties and EMT, whereas its expression is lower, although still detectable, in luminal A and B tumors [88]. Beyond EZH2, SETD2, KMT2C, and other histone methyltransferases are recurrently mutated in BC, as shown in large-scale sequencing studies [89]. 

Histone acetylation is regulated by the interplay between histone acetyltransferases and histone deacetylases. Histone acetyltransferases catalyze lysine acetylation by transferring an acetyl group from acetyl-CoA to lysine residues, generating ε-N-acetyl lysine and promoting euchromatin formation [90]. Histone acetyltransferases have been reported to function as both oncogenes and tumor suppressors in several cancer types, including BC [91]. For example, elevated P300 expression has been observed in primary BC [92]. Conversely, P300 has also been shown to increase catechol-o-methyltransferase gene expression, which reduces cell proliferation *in vitro* [93].

By contrast, histone deacetylases catalyze deacetylation through hydrolysis of acetyl groups from lysine residues [94]. Notably, histone deacetylases may exert dual roles in BC, acting either as tumor promoters or suppressors depending on cellular context, as illustrated by HDAC1. HDAC1 can suppress ER expression and its downstream genes in ER-negative BC, thereby promoting tumor growth [95]. It has also been reported to enhance BC cell proliferation and migration through upregulation of interleukin-8 signaling [96]. Conversely, HDAC1 has been shown to suppress Wnt signaling, leading to reduced migration and invasion in BC cells [97].

### 2.4 TME and Immune Modulation

In BC, TME is not merely a structural framework surrounding malignant cells, but a dynamic and continuously evolving milieu in which multiple cell types interact to influence disease progression. Alongside cancer cells, a diverse population of stromal, vascular, and immune cells contributes to tumor growth, metastatic potential, and therapeutic response. The TME includes cancer-associated fibroblasts (CAFs), tumor-associated macrophages (TAMs), myeloid-derived suppressor cells (MDSCs), regulatory T cells (Tregs), dendritic cells, and tumor-infiltrating lymphocytes (TILs), each of which plays distinct roles in modulating anti-tumor immunity. Understanding the complexity of these interactions, together with the molecular mechanisms that sustain them, is essential for developing more effective immunomodulatory strategies in BC [98,99]. In addition to cellular components, soluble factors such as cytokines (e.g., IL-6, IL-17, TGF-β) and metabolic mediators (e.g., lactate, ketone bodies) play critical roles in shaping the TME by promoting immunosuppression, angiogenesis, and metabolic reprogramming of both tumor and immune cells [100,101,102]. Tumors can exploit these regulatory networks to promote immune evasion, often through immune checkpoint pathways. Key immune checkpoint proteins involved in this process include programmed cell death-1 (PD-1), programmed cell death ligand-1 (PD-L1), and cytotoxic T-lymphocyte antigen-4 (CTLA-4), all of which have emerged as major targets in cancer immunotherapy [103]. From a clinical perspective, some components of the TME, particularly TILs, are already evaluated in routine pathology, especially in TNBC and HER2-positive tumors, where they provide prognostic information and may help predict response to chemotherapy and immunotherapy [104].

#### 2.4.1 Cancer-Associated Fibroblasts

CAFs represent one of the predominant stromal populations in breast tumors and play a central role in remodeling TME. These cells induce immunomodulatory effects through the secretion of chemokines, cytokines, and extracellular matrix components that directly and indirectly suppress anti-tumor immune responses. For example, specific CAF subsets, including the CAF-S1 subtype, recruit and expand Tregs through CXCL12 secretion, thereby enhancing immunosuppression and reducing cytotoxic T cell responses [98,105].

Single-cell transcriptomic analyses have further highlighted the heterogeneity of CAF populations in BC. Distinct subsets have been identified, including inflammatory CAF (iCAF) and matrix-modulating CAFs, each associated with different immunomodulatory profiles. Tumors enriched in iCAFs tend to display marked stromal infiltration and reduced CD8^+^ T cell activity, features that correlate with poor prognosis and radiotherapy resistance [106]. Consistently, immune gene signatures driven by iCAF-related pathways have been shown to stratify patients into high- and low-risk groups, reflecting differences in lymphocyte infiltration and macrophage phenotype [107].

Mechanistically, CAFs also modulate myeloid cell behavior. Stromal-derived factors such as MCP-1 and SDF-1 can drive the differentiation of recruited monocytes toward M2-like, immunosuppressive macrophages, thereby reinforcing protumorigenic myeloid interactions. *In vitro* and *ex vivo* analyses of BC specimens have shown that macrophages conditioned by CAFs typically express elevated IL-10 and reduced IL-12 levels, a cytokine profile that compromises effector T cell activation and enhances tumor invasiveness [108]. At present, CAF assessment is not standardized in routine clinical practice and is mainly restricted to research settings; however, its potential as a predictive biomarkers for response to immunotherapy is currently being actively explored [109].

#### 2.4.2 Tumor-Associated Macrophages

TAMs represent one of the most abundant immune cell populations in the breast TME and display remarkable phenotypic plasticity. Traditionally, TAMs are broadly classified into pro-inflammatory M1 and immunosuppressive M2 phenotypes, with M2-like TAMs becoming increasingly dominant during tumor progression and contributing to immune escape [110]. These cells secrete factors such as IL-10 and TGF-β, which suppress cytotoxic T lymphocyte (CTL) activity, promote Treg recruitment, and enhance tumor growth [111].

Recent single-cell analyses have shown that TAMs are more heterogeneous. than this simplified classification suggests. In human BC, PD-L1-positive TAMs may adopt a more immunostimulatory phenotype, support CD8^+^ T cell proliferation, and associate with better patient outcomes, whereas, PD-L1-negative TAMs tend to localize closer to tumor cells and contribute to a more immunosuppressive environment [112]. Despite extensive characterization, TAM subtyping is not routinely performed in diagnostic pathology, and its clinical utility remains investigational [113].

#### 2.4.3 Myeloid-Derived Suppressor Cells

MDSCs are a heterogeneous population of immature myeloid cells with strong immunosuppressive activities. These cells are frequently enriched in breast tumors, and their presence is often associated with advanced disease and increased metastatic potential. Preclinical BC models have shown that MDSCs inhibit T-cell proliferation through multiple mechanisms, including ARG1 expression, PD-L1 upregulation, and STAT3 activation. Collectively, these pathways impair antigen presentation and reduce CTL activity, thereby creating a TME that promotes cancer progression [114,115].

In addition to suppressing T cells, MDSCs can reprogram B cells into immunosuppressive PD-1–/PD-L1+ regulatory B cells through activation of PI3K/AKT/NF-κB signaling. The presence of these regulatory B cells in the TME has been associated with poorer clinical outcomes [108]. Currently, MDSCs are not routinely tested in clinical pathology practice, and their measurement is largely confined to experimental and translational studies [116].

#### 2.4.4 Regulatory T Cells

Tregs play a central role in maintaining immune tolerance, but tumors frequently exploit their immunosuppressive functions to suppress antitumor immunity. High infiltration of FOXP3^+^ Tregs in breast tumor stroma is generally associated with reduced effector T-cell activity and may predict adverse outcomes [117]. Tregs suppress effector T-cell responses through multiple mechanisms. They express checkpoint molecules such as CTLA-4 and secrete immunosuppressive cytokines, including IL-10 and TGF-β, collectively limiting the proliferation and function of antitumor T cells. Through these mechanisms, Tregs contribute to the immunosuppressive environment that allows breast tumors to evade immune surveillance [118,119].

#### 2.4.5 Tumor-Infiltrating Lymphocytes and Dendritic Cells

TILs, particularly CD8^+^ CTL, are the principal effector cells mediating antitumor immunity. In BC, high densities of TILs, especially in TNBC and HER2-enriched subtypes, have been associated with better responses to chemotherapy and immunotherapy [120,121]. Interestingly, chemotherapy itself can reshape the TME, altering TILs composition and the expression of immune checkpoint markers [122]. In contrast to other TME components, TILs are routinely evaluated in pathology, particularly in TNBC and HER2-positive tumors, using standardized histopathological methods (e.g., stromal TIL scoring according to international guidelines). High TIL levels are clinically meaningful, as they are associated with improved prognosis and better response to chemotherapy and immune checkpoint inhibitors [123].

Dendritic cells within the TME are essential for antigen presentation and initiating T-cell responses. However, their function can be compromised by tumor- and stromal-derived factors such as PGE_2_ and CAF-secreted TGF-β. These signals inhibit DC maturation, leading to reduced antigen presentation and impaired T-cell activation, thereby limiting the overall antitumor immune response [124,125].

#### 2.4.6 Immune Checkpoint Modulation and Therapeutic Implications

Immune checkpoints are upregulated in response to prolonged immune cell activation and function as crucial negative regulators that protect peripheral tissues from excessive damage [126]. Their activation suppresses the cytotoxic activity of immune cells, thereby maintaining the balance between self-tolerance and prevention of autoimmunity [127,128]. Tumors, however, can exploit this regulatory network to gain a survival advantage. By co-opting immune checkpoint pathways, they establish an immunosuppressive microenvironment that enables escape from immune-mediated destruction. Several checkpoint proteins have emerged as major therapeutic targets, with the most clinically advanced strategies focusing on inhibitors of PD-1, PD-L1, and CTLA-4 [103].

The PD-1/PD-L1 axis is a key immune checkpoint exploited by breast tumors to escape immune detection. PD-L1, expressed on both tumor cells and immune cells, such as TAMs, binds PD-1 on T cells, leading to T-cell exhaustion and reduced CTL activity. Blocking this pathway with immune checkpoint inhibitors can restore T-cell function and has shown clinical benefit in selected BC populations, particularly in TNBC when combined with chemotherapy [129].

CTLA-4 is another critical regulator of T-cell activation. Expressed on regulatory and anergic T cells [130], CTLA-4 competes with CD28 for binding to CD80 and CD86 on antigen-presenting cells, thereby inhibiting co-stimulatory signals necessary for T cell activation [131,132]. Relocation of CTLA-4 to the cell membrane following T-cell receptor activation, together with subsequent phosphorylation, stabilizes its inhibitory function and limits T-cell-mediated immune responses [133]. Blockade of CTLA-4 with immune checkpoint inhibitors prevents this inhibitory signaling and can restore T-cell activity [134].

#### 2.4.7 Cytokines and Metabolic Factors in the TME

In addition to the cellular components described above, soluble mediators and metabolic alterations further refine the functional landscape TME in BC, acting as critical regulators of immune cell behavior and tumor progression. Cytokines and metabolic cues operate in an interconnected manner, shaping immune dysfunction, stromal remodeling, and therapeutic resistance [135]. Recent evidence highlights that pro-inflammatory cytokines such as IL-6, TNF-α, and IL-1β are frequently elevated in BC and are strongly associated with poor clinical outcomes. IL-6, in particular, promotes tumor progression through activation of the JAK/STAT3 pathway, enhancing tumor cell survival while simultaneously expanding immunosuppressive populations, including MDSCs and Tregs [100]. Moreover, cytokines such as IL-8 and IL-17 contribute to angiogenesis and metastatic dissemination, reinforcing a pro-tumorigenic inflammatory milieu [100,136]. Importantly, cytokine signaling integrates with the immunoregulatory networks described in previous sections. For instance, TGF-β, already implicated in Treg function and CAF-mediated remodeling, acts as a central mediator of immune exclusion by limiting effector T-cell infiltration into tumor nests. This cytokine-driven stromal barrier has been associated with reduced responsiveness to immune checkpoint blockade therapies in BC [98]. Collectively, cytokine networks and metabolic adaptations act synergistically with cellular components of the TME to sustain immune dysfunction and tumor progression in BC. Targeting these interconnected pathways may enhance the efficacy of current immunotherapies and provide new opportunities for combinatorial treatment strategies. Taken together, these TME features are not only mechanistic determinants of BC progression, but also clinically relevant modifiers of immunotherapy response: immune-inflamed tumors with higher TIL content and permissive checkpoint biology are more likely to benefit from checkpoint blockade, whereas immune-excluded or immunosuppressive ecosystems characterized by CAF/TGF-β signaling, suppressive myeloid populations, and Treg enrichment provide a biological rationale for the combination strategies [137], discussed later in Section 4.3.

**Figure 1:**
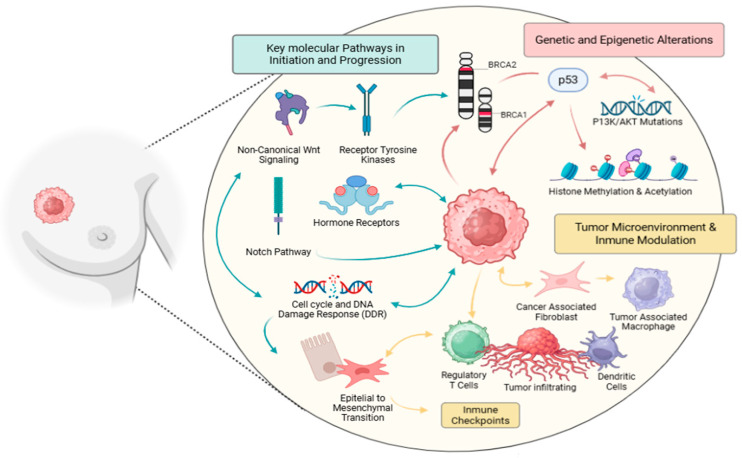
**Molecular Pathogenesis of Breast Cancer (BC).** Interconnected mechanisms coordinate BC initiation and progression: (I) genetic and epigenetic alterations (e.g., BRCA1/2, p53, PI3K/AKT mutations, and histone modifications), cell-cycle regulation, DNA damage response, and epithelial–mesenchymal transition (EMT); (II) membrane receptors (RTKs and HRs) activate key signaling pathways such as PI3K/AKT, RAS/MAPK, Wnt, and Notch, (III) tumor microenvironment, including immune and stromal cells and immune checkpoints. Abbreviations: AKT, protein kinase B; BRCA1, breast cancer susceptibility gene 1; BRCA2, breast cancer susceptibility gene 2; DDR, DNA damage response; DNA, deoxyribonucleic acid; PI3K, phosphoinositide 3-kinase; p53, tumor protein 53. Figure was created using the graphing software BioRender.

All these mechanisms mentioned above that are implicated in BC pathogenesis are graphically summarized in Fig. 1.

## 3 Diagnosis and Biomarkers

**Figure 2:**
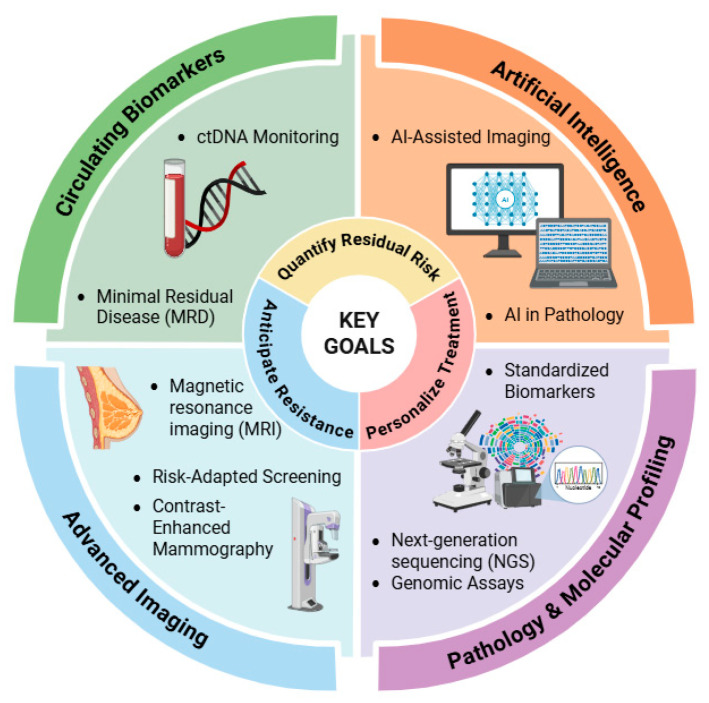
Emerging approaches in diagnosis of breast cancer. Main pillars of modern BC precision care. It integrates four domains, circulating biomarkers, AI, advanced imaging, and pathology/molecular profiling, showing how these tools contribute to three central goals: quantifying residual risk, anticipating resistance, and personalizing treatment. Figure was created using the graphing software BioRender.

BC diagnosis remains anchored in imaging-led detection followed by tissue confirmation, but the clinical objective has broadened from “confirm malignancy” to “define actionable biology early, quantify residual risk, and anticipate resistance” [138]. This evolution is driven by persistent miss rates in subgroups such as women with extremely dense breasts and those with a personal history of BC, by clinically relevant intratumor heterogeneity that can be under-sampled by a single biopsy, and by treatment landscapes that increasingly require granular biomarker assignment [139]. In practice, the most impactful emerging approaches are those that either reduce interval cancers through risk-adapted imaging pathways, or reduce biologic uncertainty through standardized pathology, genomic stratification, and longitudinal circulating biomarkers [140,141]. All of these approaches described below are graphically summarized in Fig. 2.

### 3.1 Overview of Current Diagnostic Tools: Imaging and Pathology

In contemporary pathways, imaging functions as the gatekeeper for biopsy and a first approximation of extent of disease, while pathology provides definitive diagnosis and the biomarker scaffold for initial treatment selection [142]. Screening and symptomatic workup operate as distinct clinical ecosystems [143]. In screening programs, standardized interpretation is operationalized through breast imaging-reporting and data system (BI-RADS), which harmonizes terminology and assigns explicit management recommendations across mammography, ultrasound, and magnetic resonance imaging, an essential prerequisite for auditing outcomes, calibrating recall thresholds, and integrating AI at scale [144,145].

A key contemporary inflection is the formal recognition of breast density as both a biological risk factor and an imaging limitation [146]. Under the FDA Mammography Quality Standards Act (MQSA) final rule, mammography facilities must provide standardized breast density notifications to patients and include density assessment in reports to clinicians, reinforcing density as a routine clinical variable that should trigger risk communication and, where appropriate, discussion of supplemental imaging [146,147]. This policy shift aligns with a broader move away from one-size-fits-all screening toward stratified pathways in which imaging intensity is matched to risk and detectability [148].

In the diagnostic setting, image-guided core needle biopsy is the workhorse procedure, but the clinically meaningful endpoint is no longer simply histologic classification. Pathology reports must reliably deliver predictive biomarkers that gate therapies alongside grade and other prognostic features [149]. The increasing therapeutic relevance of low-level HER2 expression has created pressure for more granular reporting, yet the 2023 American Society of Clinical Oncology/College of American Pathologists HER2 guideline update explicitly notes that “HER2-low” is not an interpretive category within the guideline framework; instead, it emphasizes assay quality and standardized interpretation to ensure reproducible results across the full scoring spectrum [149]. In parallel, biomarkers such as Ki-67 remain clinically attractive but analytically challenging; international consensus positions Ki-67 as most defensible at extreme cut-points (very low vs. very high) and underscores the importance of pre-analytical control and standardized scoring, with automation proposed as a potential route to improved reproducibility [150].

Clinically, the vulnerability of the current paradigm is the interface between imaging and pathology: discordance between suspicious imaging and benign pathology, or between heterogeneous imaging patterns and a single biomarker profile, is a recurrent source of delayed diagnosis and undertreatment [151]. This is precisely where “pathology-driven” strategies must be interpreted as workflow design: imaging that targets representative regions, pathology that reports in standardized, decision-linked formats, and multidisciplinary governance that treats discordance as a safety signal rather than an administrative inconvenience [152,153].

In practical terms, a pathology-driven approach means that the pathology report does not merely confirm malignancy, but actively structures diagnosis, risk assessment, and treatment selection. First, histologic type, grade, invasion status, lymphovascular invasion, nodal involvement, and margin status define the diagnostic category and establish baseline clinicopathologic risk [20]. Second, ER, PR, HER2, and Ki-67 translate morphology into treatment-relevant biology: HR-positive tumors are directed toward endocrine-based strategies; HER2-positive tumors toward anti-HER2 therapy; and triple-negative tumors toward chemotherapy-centered pathways, with additional biomarker testing when clinically indicated [149,150]. Third, in selected settings, pathology is extended by decision-linked biomarkers that refine escalation or de-escalation. For example, in early HR-positive/HER2-negative disease, multigene expression assays may complement routine pathology to estimate recurrence risk and chemotherapy benefit, whereas in metastatic triple-negative disease, PD-L1 testing guides eligibility for immune checkpoint blockade [154,155,156]. In parallel, stromal TIL assessment, particularly in TNBC and HER2-positive disease, can complement prognostic stratification and may help contextualize the likelihood of response to systemic therapy [157,158]. Thus, the practical value of a pathology-driven approach lies in converting tissue findings into a sequence of clinically actionable decisions rather than treating pathology as a purely descriptive endpoint.

### 3.2 Advances Liquid Biopsy and Circulating Biomarkers

Liquid biopsy in BC has matured from a general concept into a set of use-case-specific tools, with ctDNA leading the field for longitudinal monitoring and molecular residual disease (MRD) assessment [159]. In the context of MRD, liquid biopsy analyzes tumor-derived analytes in peripheral blood, including ctDNA that carries somatic mutations or other genomic alterations, and intact circulating tumor cells that can be molecularly profiled to detect residual tumor biology after therapy. Hence, molecular classification informs MRD monitoring by using ctDNA and CTC analyses to capture subtype-specific genomic features, enabling personalized risk stratification, surveillance, and insights into tumor heterogeneity and therapy resistance [160].

The biological constraint that shapes all applications is tumor fraction: early-stage cancers and minimal residual disease shed little ctDNA, so performance hinges on assay sensitivity, sampling frequency, and whether the approach is tumor-informed (tracking patient-specific variants derived from tumor sequencing) versus tumor-agnostic [159]. Tumor-informed assays based on individualized mutation panels derived from the primary tumor improve detection of MRD by focusing on known somatic alterations, while broader genomic or epigenetic profiling can complement subtype-specific information and capture dynamic changes in tumor biology [161].

The strongest near-term clinical value lies in risk stratification and monitoring rather than population screening. MRD detection after curative-intent therapy is prognostically powerful, but its clinical actionability is still being defined [162]. The c-TRAK TN trial illustrates both the promise and the current limitation of ctDNA-triggered intervention in early TNBC: ctDNA positivity identified a very high-risk subgroup, yet many patients already had overt metastatic disease at the time of ctDNA detection, implying that earlier initiation of testing and/or more frequent or more sensitive assays may be required for MRD-guided escalation to meaningfully shift outcomes [163]. This has direct implications for clinical protocol design: ctDNA should be conceptualized as an early warning layer that must be paired with rapid confirmatory imaging and a predefined intervention pathway to avoid “prognosis without consequence”.

In neoadjuvant and post-neoadjuvant settings, ctDNA dynamics are increasingly positioned as a quantitative biomarker that complements imaging response and pathologic response. Studies using tumor-informed assays show that ctDNA can refine risk stratification among patients with therapy-resistant residual disease and may provide earlier signals of relapse risk than conventional surveillance, particularly in those with high residual cancer burden [164,165]. The clinical attraction here is practical: ctDNA offers a minimally invasive way to follow tumor biology across time, potentially identifying patients who should be prioritized for escalation trials or intensified surveillance.

From a clinical pathway perspective, ctDNA currently appears most relevant in three scenarios: post-neoadjuvant or post-adjuvant risk stratification after curative-intent therapy, longitudinal MRD surveillance in prospective protocols, and molecular profiling in advanced disease when tissue is unavailable or repeated sampling is impractical. However, current guideline-level recommendations remain cautious. ESMO states that molecular residual disease or molecular relapse detection using ctDNA cannot currently be recommended in routine clinical practice because evidence of clinical utility is still lacking. Therefore, a positive ctDNA result should not yet be considered an automatic trigger for therapeutic escalation outside a predefined clinical protocol, but rather a signal that may justify confirmatory imaging, multidisciplinary review, and consideration of trial enrollment [162,166].

Circulating biomarkers beyond ctDNA (circulating tumor cells, cfRNA, methylation, extracellular vesicles) remain important for the future trajectory of early detection and mechanistic stratification, but most are not yet positioned for routine, guideline-driven decision-making in average-risk screening [167]. Another key barrier to broader implementation is assay harmonization. Recent consensus work emphasizes that reliable ctDNA testing requires rigorous standardization across the pre-analytical, analytical, and post-analytical phases, including blood collection, cfDNA isolation, assay validation, and result interpretation [168]. In addition, cost-effectiveness remains insufficiently established for routine BC MRD monitoring, particularly outside highly selected clinical contexts. Thus, before ctDNA can be fully integrated into standard BC pathways, prospective studies must demonstrate not only prognostic value, but also reproducibility, actionable clinical benefit, and economic justification [168].

Overall, ctDNA in BC should currently be framed as a high-potential translational tool with strong prognostic relevance, but not yet as a universally standardized or guideline-endorsed routine instrument for MRD-directed treatment decisions.

### 3.3 Advanced Imaging Techniques

Advanced breast imaging is best understood as functional augmentation of anatomic screening, deployed to mitigate known failure modes, especially masking in dense breasts and reduced sensitivity in women with a prior BC [169]. Contrast-enhanced mammography (CEM) has emerged as a pragmatic functional modality that can approximate some of the lesion conspicuity advantages of magnetic resonance imaging while operating within mammography workflows [170]. In a prospective trial interim analysis, adding CEM to digital breast tomosynthesis in women with a personal history of BC addressed a clinically important scenario: digital breast tomosynthesis alone can be inadequate in this higher-prevalence group, who also experience higher interval cancer rates [171]. In fact, certain imaging features have been associated with subtype-specific, suggesting an imaging phenotype that complements molecular classification. This may effectively predict BC molecular subtypes preoperatively, potentially informing of risk-adapted screening or surveillance strategies [172]. CEM enhancement patterns and quantitative measures (e.g., degree of contrast uptake) have been linked in some studies to more aggressive molecular phenotypes, such as HER2-positive and TNBC subtypes, which tend to exhibit stronger vascularity and earlier contrast wash-in/out [173]. This is a clinically persuasive positioning for CEM: not as a universal replacement for magnetic resonance imaging, but as a scalable functional add-on where magnetic resonance imaging access is constrained or where rapid problem solving is required [174]. When combined with clinical and molecular data, such imaging can contribute to more nuanced risk models that align with molecular classification frameworks currently used in precision oncology.

Abbreviated magnetic resonance imaging protocols are similarly motivated by feasibility. The goal is to preserve magnetic resonance imaging’s sensitivity while reducing acquisition and interpretation burdens, enabling broader deployment for supplemental screening. The clinical debate is no longer whether supplemental imaging finds more cancers, it does, but which modality offers the best balance of yield, harms, and feasibility for specific risk strata. A trial in women with dense breasts and negative mammography reported substantially higher cancer detection with abbreviated magnetic resonance imaging and CEM compared with automated ultrasound, strengthening the rationale for prioritizing functional imaging over ultrasound-only supplementation when resources permit [175].

Guidelines are increasingly explicit about dense-breast strategy. The European Society of Breast Imaging (EUSOBI) recommends offering screening magnetic resonance imaging to women with extremely dense breasts, citing reduced mammographic sensitivity and evidence from the Dutch DENSE trial; this provides a strong European consensus anchor for risk-adapted pathways in dense tissue [176]. Clinically, these recommendations have operational consequences: magnetic resonance imaging capacity planning, patient selection criteria, and protocols for handling the downstream increase in incidental findings and biopsies [177].

Implementation considerations are central and should be stated plainly. CEM requires iodinated contrast (allergy and renal assessment) and adds radiation relative to standard mammography (protocol-dependent) [178]. Magnetic resonance imaging requires gadolinium considerations, longer workflows, and often constrained capacity. Across modalities, the clinically relevant endpoint is not detection per se, but reduction in interval cancers and stage shift without unacceptable increases in false positives, overdiagnosis, or inequities in access [179].

### 3.4 Molecular and Genomic Profiling

Molecular profiling now sits on the critical path of “diagnosis-to-treatment,” particularly for early-stage HR-positive, HER2-negative disease where escalation and de-escalation decisions hinge on recurrence risk and expected chemotherapy benefit. Multigene expression assays have the most mature evidence base. TAILORx established that many patients with intermediate 21-gene recurrence scores can safely avoid chemotherapy, while RxPONDER extended the framework to patients with 1–3 positive nodes and demonstrated clinically important effect modification by menopausal status [154,156]. The MINDACT trial’s long-term follow-up supports omission of chemotherapy in clinically high-risk but genomically low-risk patients, with small absolute benefit estimates that reinforce the principle of targeted de-escalation [155]. These trials collectively justify positioning gene-expression profiling as part of a pathology-driven therapeutic strategy: it is an extension of diagnostic classification into treatment utility.

Germline testing is increasingly integrated into diagnostic planning because results can determine eligibility for targeted systemic therapies (for example, PARP inhibitors in appropriate settings) and shape risk management for patients and families. The 2024 ASCO–SSO guideline expands and clarifies who should be offered BRCA1/2 testing, providing a contemporary consensus basis for upstream incorporation into care pathways [180].

Broader tissue next-generation sequencing and ctDNA-based profiling are most firmly established in advanced disease and in trial contexts, but their relevance to “early diagnosis and therapeutic strategies” lies in targeted situations: resolving ambiguous receptor biology, identifying actionable alterations when tissue is limited, and enabling serial assessment of resistance mechanisms [181].

### 3.5 AI in Diagnosis

AI is transitioning from retrospective performance reporting to prospective, workflow-level evaluation in screening programmes. The most credible near-term model is “AI as a second reader and triage layer,” not AI as an autonomous diagnostic authority [182]. A randomized trial evaluated AI-supported mammography screening with outcomes including interval cancer rates and showed non-inferior interval cancer outcomes compared with standard double reading, while supporting favorable screening performance metrics [175]. Complementing trial evidence, a nationwide real-world implementation study reported that AI-supported double reading was associated with higher cancer detection without negatively affecting recall rate, directly addressing the operational concern that improved sensitivity might come at the cost of unacceptable false positives [183]. Clinically, these results support deployment scenarios where AI reallocates human expertise: low-risk examinations can be streamlined, while higher-risk or suspicious cases receive intensified human review.

In pathology, AI’s most clinically defensible roles in the near term are quality assurance, biomarker support, and heterogeneity detection rather than replacing established immunohistochemistry and molecular assays. A study demonstrated deep learning prediction of ER, PR, and ERBB2 status from H&E slides and explicitly evaluated utility in scenarios such as second-read quality assurance and addressing intratumor heterogeneity [184]. In parallel, foundation-model approaches in computational pathology are accelerating the development of robust slide-based tools for diagnosis, prognosis, and biomarker prediction, which is likely to lower the barrier to developing clinically useful models across institutions [185].

Both radiology and pathology AI are sensitive to domain shift (scanner vendors, acquisition protocols, staining and fixation, population prevalence). AI tools should be implemented with continuous monitoring, calibration, and clearly bounded intended use (triage, second read, quality control), rather than as generic “improvers” of diagnostic accuracy.

#### Integration of AI and Multi-Omics Approaches in Diagnosis and Prognosis

Multimodal AI is increasingly framed as a route to more robust and clinically meaningful inference because it contextualizes molecular features within anatomical and clinical frameworks rather than treating each data stream in isolation [186]. In BC, the clinically plausible “integrated stack” is already visible: density- and risk-informed screening policies, AI-supported mammography workflow optimization, standardized pathology and biomarker reporting, selective use of gene-expression assays to guide adjuvant intensity, and longitudinal ctDNA monitoring in high-risk or trial-driven contexts [187]. The key translational benchmark is prospective utility: integration matters clinically only when it reduces interval cancers, improves stage shift, or enables earlier, evidence-based escalation or de-escalation decisions, outcomes that can be measured and audited in real-world systems, not merely inferred from retrospective gains.

Despite these advances, several critical gaps must be addressed before AI can be safely adopted for routine clinical practice. First, AI models require high performance and stability for clinical use, but their performance often declines when applied to new, heterogeneous data, limiting widespread adoption [173]. Second, current AI methods for medical image analysis still struggle with issues such as integrating multi-modal data, dealing with imbalanced datasets, and the lack of clear interpretability and uncertainty quantification [188]. Third, integration with existing clinical workflows and health infrastructure remains an unresolved challenge. Finally, ethical and legal challenges must be considered. For instance, those related to data privacy and security, unclear liability in case of errors, the need for regulatory frameworks adapted to dynamic systems, and the risk of overreliance that may undermine clinical judgment and patient-centered care [189].

## 4 Therapeutic Advances in BC

### 4.1 Standard Therapies: Surgery, Radiotherapy, Endocrine and Chemotherapy

BC treatment is complex and requires a multidisciplinary approach that integrates tumor burden, disease stage, molecular subtype, and patient-related factors. The traditional therapeutic modalities of BC include surgery, radiotherapy, chemotherapy and endocrine therapy, with treatment schedules typically coordinated across multiple specialties [190]. Available systemic treatment options are determined by the stage, from I (small and anatomically defined tumors) to IV (metastatic disease), and by tumor subtype, including HR-positive/HER2-negative, HER2-positive, and TNBC [191].

For non-metastatic BC, surgery remains the cornerstone of treatment with the main objective of achieving local disease control and reducing the risk of recurrence. Local surgical therapy may be preceded by neoadjuvant systemic therapy to reduce the primary tumor size, increase the likelihood of breast-conserving surgery, and decrease the need for axillary lymph node dissection; postoperative radiotherapy may then be indicated according to surgical and pathological findings. In contrast, systemic therapy is the preferred treatment for metastatic BC, in which symptom palliation and prolongation of survival are the principal goals, while surgery is generally reserved for selected cases, mainly for palliative purposes [190,191]. The administration of systemic therapy before surgery is known as neoadjuvant therapy. Although this strategy was initially developed for inoperable BC, it is now considered appropriate in several clinical settings, including inflammatory BC and patients in whom the assessment of residual disease after systemic therapy may guide subsequent treatment decisions [192].

Radiotherapy, which uses ionizing radiation to eradicate residual malignant cells, is routinely administered after breast-conserving surgery to reduce the risk of local recurrence and improve local control, traditionally through daily treatments over three to six weeks, although shorter regimens are increasingly used [193].

Systemic therapy may also be used as the main treatment, in combination with local therapies, or after surgery. When administered after surgery, it is referred to as adjuvant therapy. The rationale for adjuvant treatment is to eradicate micrometastatic disease, as BC cells may disseminate from the primary tumor at an early stage and persist in distant tissues in a clinically undetectable state for prolonged periods [194]. Chemotherapy consists of cytotoxic agents, including anthracyclines and taxanes, that interfere with cell division and remains a key strategy for reducing recurrence in many patients with stage I–III BC. It also continues to represent a major systemic treatment backbone in TNBC. Adjuvant chemotherapy may be administered alone or in combination with radiotherapy, depending on the clinical scenario [191,195]. Endocrine therapy, in turn, is the principal systematic treatment HR-positive/HER2-negative BC, as it suppresses estrogen-driven tumor growth [190]. Standard endocrine treatment typically involves daily oral therapy with anti-estrogen agents, such as tamoxifen or aromatase inhibitors, for 5 to 10 years, with the specific regimen determined by menopausal status and recurrence risk [191].

### 4.2 Targeted Therapies and Precision Medicine: PARP Inhibitors, HER2 Inhibitors, CDK4/6 Inhibitors

In recent years, precision oncology has significantly transformed BC management by enabling treatments selection to specific molecular alterations within tumors. Among the most clinically relevant strategies are PARP inhibitors, HER2-targeted therapies, and CDK4/6 inhibitors [196].

PARP inhibitors, such as olaparib and talazoparib, are used primarily in selected patients with germline BRCA1- or BRCA2-mutated, HER2-negative BC, as these tumors frequently exhibit homologous recombination deficiency, rendering them particularly sensitive to PARP inhibition through synthetic lethality. Mechanistically, these agents block PARP-mediated repair of single-strand DNA breaks, leading to the accumulation of genomic damage and ultimately tumor cell death. PARP inhibitors have demonstrated significant improvement in progression-free survival compared with standard chemotherapy in patients with BRCA-mutated, HER2-negative metastatic BC [197].

In HER2-positive BC, amplification or overexpression of the HER2 oncogene promotes tumor proliferation and survival. Accordingly, the development of HER2-directed therapies, including monoclonal antibodies such as trastuzumab and pertuzumab, as well as antibody-drug conjugates such as trastuzumab deruxtecan, has markedly improved clinical outcomes in this subtype. Furthermore, dual blockade with trastuzumab and pertuzumab has demonstrated significant improvements in response rates and survival [196].

CDK4/6 inhibitors, including palbociclib, ribociclib, and abemaciclib represent another major advance in targeted therapy, particularly for HR-positive/HER2-negative BC. Dysregulation of the cyclin D–CDK4/6–retinoblastoma pathway leads to uncontrolled cell-cycle progression from the G1 to S phase in many BCs. These agents selectively inhibit CDK4/6 activity, restoring cell-cycle control and suppressing tumor proliferation. When combined with endocrine therapy, CDK4/6 inhibitors have become the standard of care for advanced HR-positive/HER2-negative BC and significantly prolong progression-free and overall survival compared with endocrine therapy alone [197].

Collectively, these three therapy options exemplify the paradigm shift toward precision medicine in BC treatment: targeting specific oncogenic drivers or vulnerabilities identified through molecular profiling to improve patient outcomes and reduce reliance on non-specific cytotoxic chemotherapy.

### 4.3 Immunotherapy and Combination Strategies

The therapeutic role of immunotherapy in BC is best understood as a clinical extension of the TME framework described in Section 2.4. Tumors with a more inflamed immune contexture, particularly subsets of TNBC, may be more likely to benefit from immune checkpoint blockade, whereas immune exclusion and suppressive stromal–myeloid interactions can limit efficacy and contribute to resistance. Accordingly, current combination strategies are designed not only to activate antitumor immunity, but also to remodel the TME. In this context, combinations of immune checkpoint inhibitors with chemotherapy, PARP inhibitors, CDK4/6 inhibitors, or radiotherapy are being investigated to increase antigen release, enhance T-cell priming, overcome stromal or myeloid-mediated suppression, and broaden the proportion of patients who derive clinical benefit from immunotherapy [198].

This microenvironment-based rationale has been particularly relevant in TNBC, a subtype characterized by the absence of ER, PR, and HER2 expression. TNBC often exhibits a higher tumor mutational burden, greater genomic instability, and increased immune-cell infiltration than other BC subtypes, which may render it more responsive to immune checkpoint blockade. In this context, immune checkpoint inhibitors targeting the PD-1 axis such as pembrolizumab, have demonstrated clinically meaningful benefit. Their combination with chemotherapy has been associated with improved clinical outcomes compared with chemotherapy alone in selected patient populations [199,200].

Overall, these multimodal strategies reflect an evolving therapeutic paradigm in which immunotherapy is increasingly integrated with other targeted and conventional treatments to achieve more durable responses and broaden the clinical benefit of immune-based therapies in BC management [198,200].

### 4.4 Challenges in Treatment Resistance and Tumor Heterogeneity

Despite major therapeutic advances, tumor heterogeneity and treatment resistance remain two of the principal barriers to effective BC management. Heterogeneity in BC exists at both intertumoral, reflecting differences between tumors in different patients, and the intratumoral, where multiple genetically and phenotypically distinct subclonal populations may coexist within a single tumor. These differences arise from a complex interplay of genetic mutations, epigenetic alterations and environmental factors, ultimately leading to marked variability in tumor behavior and therapeutic response [201]. Advances in genomic sequencing technologies have revealed that breast tumors frequently evolve under therapeutic pressure, allowing resistant clones to expand while sensitive cell populations are eliminated [201,202]. Furthermore, biological processes such as EMT, metabolic reprogramming, and the persistence of cancer stem-like cells have been associated with increased metastatic capacity and resistance to both conventional and targeted treatments [202].

This molecular heterogeneity also plays a central role in resistance to targeted therapies. For instance, HER2-enriched BC may display heterogeneous HER2 expression within different regions of the same tumor or between primary and metastatic lesions, a phenomenon known as spatial heterogeneity. This variability can reduce the effectiveness of HER2-targeted therapies because not all tumor cells express sufficient levels of the target receptor [203]. Similarly, resistance to endocrine therapy or CDK4/6 inhibitors in HR-positive BC has been linked to diverse molecular alterations and clonal evolution. Recent single-cell transcriptomic studies have demonstrated that biomarkers associated with resistance to CDK4/6 inhibitors show significant variability across tumor cell populations, suggesting that resistant clones may already exist prior to treatment and expand during therapy [204]. Moreover, TME further contributes to creating resistance by providing favorable conditions and generating protective niches for tumor cell survival and immune evasion [205].

Together, these findings underscore the importance of understanding tumor evolutionary dynamics, are determined not only by tumor-cell genetics but also by the broader cellular and stromal context, in order to design more effective and durable therapeutic strategies.

### 4.5 Radiation Therapy Innovations and Surgical Advancements

Traditionally, adjuvant whole-breast irradiation following breast-conserving surgery has been a standard component of therapy, significantly reducing the risk of local recurrence and improving long-term survival in patients with early-stage BC. In recent years, innovations in radiotherapy planning and delivery, including three-dimensional conformal radiotherapy and intensity-modulated radiotherapy, have improved the ability to precisely target tumor beds while minimizing radiation exposure to surrounding normal tissues such as the heart and lungs. These approaches allow more homogeneous dose distribution and have been associated with reduced treatment-related toxicity and improved cosmetic outcomes compared with conventional radiotherapy approaches [206,207]. Accelerated partial breast irradiation (APBI) is a treatment approach which consists in targeting only the tumor bed rather than the entire breast, based on the observation that most local recurrences occur near the original tumor site. Clinical trials have shown that APBI can provide comparable local control to whole-breast irradiation in selected low-risk patients while reducing radiation exposure to healthy tissues [208]. Another major innovation in BC radiotherapy is the development of hypofractionated radiotherapy schedules, which deliver higher doses per fraction over a shorter treatment period. More recently, ultra-hypofractionated regimens have shown promising results, further shortening treatment time while maintaining similar efficacy and safety profiles [209].

Surgical management of BC has evolved significantly over the past decades, shifting from highly radical procedures toward more conservative and personalized approaches, with breast-conserving surgery becoming the preferred surgical option for many patients when technically feasible. In parallel, advances in imaging techniques and intraoperative margin assessment have improved the precision of tumor excision, reducing the need for re-operations and improving surgical outcomes [210]. Another major advancement in BC surgery has been the development of sentinel lymph node biopsy which has largely replaced routine axillary lymph node dissection, significantly reducing complications such as lymphedema, pain, and shoulder dysfunction while maintaining reliable staging accuracy [211]. Currently, surgical interventions are also focused on improving cosmetic outcomes and patient quality of life through techniques such as oncoplastic surgery and nipple-sparing mastectomy. Oncoplastic breast surgery combines principles of oncologic tumor resection with plastic surgery techniques to allow wider tumor excision while maintaining breast symmetry and aesthetics [212].

### 4.6 Multimodal and Personalized Treatment Strategies

Recent technological advances, particularly single-cell RNA sequencing and other multi-omics approaches, have significantly improved the understanding of cellular diversity, transcriptional plasticity, and TME interactions in BC. However, clonal heterogeneity is more directly defined by DNA-level alterations; therefore, single-cell DNA sequencing or integrated multi-omic approaches are better suited to characterize clonal architecture and tumor evolution [213,214]. These techniques allow researchers to analyze gene expression patterns at the individual cell level, revealing previously unrecognized subpopulations of tumor and immune cells, thus providing insights into how these populations evolve during treatment [215]. By identifying resistant cell populations early and monitoring tumor evolution over time, these technologies may help guide the development of more effective treatment strategies, including rational combination therapies and adaptive treatment approaches aimed at preventing the emergence of resistant clones [204]. 

Integrated diagnostic–therapeutic platforms and biomarker-driven approaches have become central to the implementation of precision medicine in BC, enabling more individualized treatment strategies based on the molecular characteristics of each tumor. Advances in genomic technologies, including next-generation sequencing and multigene expression assays, have significantly improved the ability to stratify patients according to recurrence risk and predicted response to therapy [156].

Patient-specific treatment regimens are therefore increasingly designed by combining traditional clinicopathological factors with molecular biomarkers and genomic data. Established biomarkers such as ER, PR, and HER2 status continue to guide the use of endocrine therapies and HER2-targeted treatments, while newer biomarkers, including PD-L1 expression, homologous recombination deficiency, and ctDNA, are expanding the range of patients who may benefit from immunotherapy or DNA-damage–targeted therapies [199,216]. Liquid biopsy approaches, particularly ctDNA analysis, are also emerging as valuable tools for monitoring tumor evolution, detecting minimal residual disease, and identifying resistance mutations during treatment [217].

**Figure 3:**
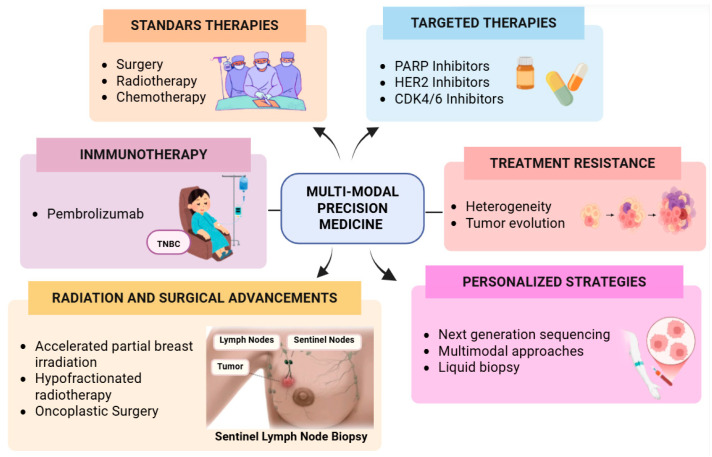
Overview of therapeutic approaches in breast cancer. It presents the relationship between standard therapies, targeted therapies, immunotherapy, radiation and surgical advances, treatment resistance, and personalized strategies. Overall, the image emphasizes that BC management increasingly depends on combining conventional treatments with molecularly guided and individualized approaches to address tumor heterogeneity and improve outcomes. Figure was created using the graphing software BioRender.

Finally, the rapid advancement of BC diagnosis and treatment highlights the need for timely, evidence-based, and context-specific national guidelines to ensure appropriate and equitable care. Inadequate regulation may result in the adoption of costly therapies with limited clinical value in certain settings. Therefore, effective BC management depends not only on therapeutic innovation but also on the integration of clinical guidelines with broader health-policy frameworks to optimize resource allocation and maximize patient benefit [218]. An overview of the previously mentioned therapeutic approaches in BC is provided in Fig. 3.

## 5 Survivorship Care

### 5.1 Long-Term Effects and Recurrence-Aware Survivorship

The growing number of BC survivors has shifted part of the clinical focus from short-term disease control to long-term survivorship. For many patients, survivorship extends well beyond the completion of active treatment and includes persistent physical, psychological, social, and economic consequences that can substantially affect quality of life [219]. Common long-term or late effects include fatigue, chronic pain, lymphedema, sleep disturbances, cognitive complaints, osteoporosis, sexual dysfunction, anxiety, depression, and fear of cancer recurrence. These burdens are compounded in some patients by financial toxicity, work-related disruption, and difficulties in social and family reintegration, reinforcing the need for survivorship care models that are multidisciplinary, longitudinal, and patient-centered [220,221].

Survivorship care must also account for the ongoing risk of recurrence, which is not uniform across patients. Follow-up needs vary according to age at diagnosis, stage, molecular subtype, treatment exposure, and comorbidity burden. Importantly, recurrence patterns differ biologically across subtypes. In HR-positive disease, the risk of distant recurrence may persist well beyond the first 5 years after diagnosis and endocrine therapy [222]. Recent long-term clinical data indicate that patients with HR-positive BC have an estimated 10% to 40% risk of distant metastasis that persists beyond the initial 5 years of adjuvant endocrine therapy, supporting the concept that this subtype is characterized by a prolonged tail of recurrence risk rather than an exclusively early hazard period. This temporal pattern has direct implications for survivorship counselling, endocrine therapy adherence, bone and cardiovascular monitoring, and the development of risk-adapted surveillance strategies [222]. By contrast, HR-negative tumors tend to show a more concentrated early recurrence pattern. These subtype-specific recurrence trajectories reinforce that survivorship care should not be designed as a uniform post-treatment package, but rather as a personalized, longitudinal care model aligned with residual biological risk [220,222]. HR-positive disease may recur many years, or even decades, after diagnosis, whereas HR-negative tumors tend to have a more concentrated early recurrence pattern. This temporal heterogeneity has important implications for surveillance intensity, survivorship counselling, and the design of risk-adapted follow-up strategies [220,223,224].

### 5.2 Follow-Up Care and Care Coordination

Survivorship care should extend beyond general follow-up recommendations and be organized as a coordinated, risk-adapted, and patient-centered model of long-term care. In clinical practice, follow-up after BC should not be limited to recurrence surveillance alone, but should also include assessment and management of treatment-related toxicities, adherence to long-term therapies, psychosocial needs, functional recovery, and comorbidity burden [220]. This is particularly relevant in BC survivors because the consequences of treatment may persist for years and often involve multiple domains, including fatigue, cognitive complaints, lymphedema, pain, menopausal symptoms, sexual dysfunction, bone loss, cardiovascular risk, and fear of recurrence [224].

A practical survivorship framework should therefore integrate several components. First, surveillance strategies should be individualized according to recurrence risk, biological subtype, age, and prior treatment exposure, rather than applied uniformly across all survivors [225]. Second, follow-up care should include active monitoring of endocrine therapy tolerance and adherence in patients with HR-positive disease, given the importance of prolonged treatment exposure in reducing late recurrence risk. Third, survivorship pathways should include systematic evaluation of long-term toxicity domains such as bone health, cardio-oncology, cognition, emotional distress, and return-to-work difficulties. Fourth, communication between oncology teams, primary care professionals, rehabilitation services, psycho-oncology, and other specialists should be strengthened to avoid fragmented care and delayed management of persistent symptoms [225].

In this context, care coordination becomes a central component of survivorship rather than an administrative add-on. Shared-care models may be particularly valuable for the growing population of BC survivors, especially when specialist resources are limited or when long-term needs extend beyond the traditional boundaries of oncology practice [226,227]. Accordingly, survivorship care plans should not only summarize prior treatment, but also define follow-up responsibilities, expected late effects, supportive care needs, and indications for re-referral. Such an approach may improve continuity of care, facilitate early detection of clinically relevant problems, and better align long-term management with the heterogeneous needs of BC survivors [226,227].

### 5.3 Practical Research Entry Points in Survivorship Care

Future work in BC survivorship should move beyond broad calls for patient-centered care and prioritize pragmatic, testable research questions. Recent evaluations of survivorship guidance show that many recommendations remain strong on surveillance but weaker on how to operationalize long-term follow-up, symptom management, care coordination, and implementation across diverse health systems. Accordingly, survivorship research should increasingly focus on interventions that can be prospectively evaluated in routine practice, rather than only describing unmet needs [220].

A first priority is the development and validation of risk-adapted follow-up pathways. Survivorship care should not be delivered as a uniform package for all patients, and future studies should test whether clinicopathological variables, treatment exposure, comorbidity burden, patient-reported outcomes, and, where appropriate, emerging biomarkers can be integrated into follow-up models that are both safe and scalable. In parallel, comparative studies are needed to determine when oncology-led care, shared-care models, or primary care–integrated survivorship pathways are most effective. Recent implementation work suggests that nurse-enabled and shared-care follow-up models for early BC are feasible and can be delivered safely, supporting this area as a concrete research direction rather than a theoretical proposal [226,228,229].

A second priority is improving adherence and tolerability of long-term endocrine therapy in HR-positive disease. This is a particularly relevant survivorship research target because suboptimal adherence to adjuvant endocrine therapy has been associated with higher recurrence risk, lower quality of life, and greater healthcare burden. Current pragmatic trials are now testing patient-centered interventions specifically designed to improve endocrine therapy adherence and cost-effectiveness, indicating that future survivorship studies should not only measure persistence with therapy, but also address symptom burden, communication, and supportive care strategies that help patients remain on treatment [230,231].

A third priority is the integration of structured symptom monitoring, self-management support, and patient-reported outcomes into survivorship pathways. Digital supportive-care platforms for BC survivors are increasingly being developed to address physical and psychosocial sequelae, improve access to evidence-based behavioral interventions, and enhance patient engagement. Future studies should therefore evaluate whether electronic patient-reported outcome systems and digital self-management tools can improve symptom control, quality of life, and care responsiveness in survivorship settings, while also addressing implementation barriers such as equity of access, digital literacy, and integration into routine care [225,232].

Finally, survivorship research should broaden its outcome framework beyond recurrence and survival alone. Persistent issues such as fear of recurrence, cognitive symptoms, sexual dysfunction, lymphedema, bone health, cardiovascular risk, and return-to-work difficulties should be treated as core survivorship endpoints rather than secondary considerations. Future research should incorporate standardized patient-reported, functional, and implementation outcomes to identify which survivorship interventions are clinically meaningful, sustainable, and equitable across different survivor populations [233,234,235].

## 6 Challenges and Future Directions across the BC Continuum

### 6.1 Biological Complexity, Dormancy, and Late Recurrence

Future progress in BC care will likely depend on integrating biological precision with health-system precision. Personalized medicine is expected to move beyond static subtype classification toward dynamic, longitudinal profiling of tumor evolution. However, one of the unresolved biological challenges is that BC progression is not driven only by detectable active disease, but also by dormant residual cell populations that may persist in a clinically silent state and later reactivate. Recent evidence indicates that hypoxia, extracellular matrix remodeling, therapy-induced stress, and immune interactions may support the survival of dormant BC cells, thereby contributing to late recurrence. In parallel, increasing recognition of intertumoral and intratumoral heterogeneity reinforces that breast tumors are evolving systems rather than fixed biological entities. Future mechanistic studies should therefore focus not only on identifying additional molecular alterations, but also on clarifying how clonal evolution, microenvironmental adaptation, and tumor-cell plasticity interact over time to shape recurrence risk and treatment response [236,237].

### 6.2 Diagnostic Translation: ctDNA, Multimodal Biomarkers, and AI

A key challenge is the incomplete translation of emerging biomarkers into routine care. Although multi-omics profiling, liquid biopsy, and single-cell technologies are rapidly expanding the biological understanding of BC, their clinical implementation is still limited by assay standardization, analytical reproducibility, external validation, regulatory uncertainty, and patient heterogeneity. Similar translational gaps affect biomarker-driven surveillance and minimal residual disease monitoring, where promising signals have not yet been fully converted into harmonized, widely deployable clinical pathways. As a result, there remains a disconnect between technological innovation and real-world clinical utility, particularly outside highly specialized centers. Future strategies should combine established public-health measures with emerging tools such as risk-based screening, molecular residual disease monitoring, and blood-based biomarkers, while ensuring that these technologies are introduced in a clinically meaningful and equitable manner [137,238,239,240]. In particular, ctDNA-based MRD detection should currently be framed as a promising but not yet routine tool. ESMO recommendations state that molecular residual disease or molecular relapse detection using ctDNA cannot currently be recommended for routine clinical practice because clinical utility has not yet been established. In addition, recent consensus work emphasizes that reliable ctDNA implementation requires standardization across the pre-analytical, analytical, and post-analytical phases, including blood collection, cfDNA isolation, assay validation, quality control, and result interpretation. Thus, future progress in diagnostic translation will depend not only on increasing analytical sensitivity, but also on demonstrating that ctDNA-guided decisions improve meaningful patient outcomes in reproducible clinical workflows [166,168].

Earlier detection must remain a parallel priority. At the population level, reducing advanced-stage presentation requires not only improved screening where appropriate, but also stronger systems for symptom recognition, referral, pathology, and diagnostic resolution. A similar translational caution applies to multimodal AI. Although AI-supported models may improve imaging interpretation, pathology workflows, and multimodal risk estimation, their broader implementation is still limited by incomplete external validation, reduced generalizability across settings, data heterogeneity, interpretability challenges, and practical barriers to integration into routine care. Accordingly, future diagnostic innovation should be judged not only by technical performance, but also by prospective multicenter validation, clinical utility, interoperability, and context-specific implementation [241,242,243].

### 6.3 Therapeutic Adaptation, Resistance, and Precision Treatment

Another major encounter is the persistence of treatment resistance. Personalized medicine may enable more accurate risk stratification, earlier identification of resistance, adaptive treatment selection, and more individualized survivorship plans. However, their clinical value will depend on prospective validation, interoperability across platforms, and demonstration of benefit in diverse patient populations. From a therapeutic perspective, resistance should be understood not only as the consequence of isolated acquired mutations, but also as the product of epigenetic remodeling, tumor heterogeneity, immune-regulatory adaptation, stromal protection, and epithelial–mesenchymal plasticity. These mechanisms allow tumor cells to evade chemotherapy, endocrine therapy, targeted agents, and, in selected settings, immunotherapy. Therefore, future treatment strategies should increasingly incorporate serial molecular assessment, rational combination regimens, and adaptive therapeutic models that account for clonal evolution and residual disease biology over time, rather than relying exclusively on one-time baseline biomarker assignment [236].

### 6.4 Future Directions in BC Care: Equity, Implementation, and Governance

Despite major advances in diagnosis and therapy, substantial challenges remain in the global management of BC. One of the most pressing is inequality in access to timely diagnosis and effective treatment. Marked disparities persist across and within countries, with higher proportions of metastatic disease at diagnosis, greater proportions of unknown stage, and poorer survival outcomes in low- and middle-income settings, older populations, and socioeconomically disadvantaged groups. These differences reflect unequal access to awareness programs, diagnostic pathways, pathology services, imaging, treatment completion, and longitudinal follow-up. The World Health Organization’s Global BC Initiative has therefore prioritized three system-level actions: health promotion and early detection, timely diagnosis, and comprehensive BC management [2,5]. Finally, advances in BC care will have limited impact unless they are accompanied by broader global access to effective diagnosis, treatment, and survivorship services. Future policy efforts should therefore prioritise scalable implementation of evidence-based care pathways, workforce training, patient navigation, access to essential medicines and pathology infrastructure, and survivorship programs that extend beyond the acute treatment period. In this sense, the future of BC management lies not only in increasingly sophisticated technologies, but also in ensuring that these innovations are translated into equitable benefits across different health systems and populations. The WHO implementation framework sharpens this agenda by linking system strengthening to measurable targets and operational readiness, emphasizing that durable progress requires not only innovation, but also diagnostic capacity, treatment completion, referral systems, and survivorship infrastructure. This means that future research and policy should place greater emphasis on implementation science, affordability, and health-system integration, especially in settings where advanced diagnostic and therapeutic tools may otherwise remain inaccessible [137,238,239,240].

Ethical considerations are also becoming increasingly relevant as AI, genomics, and liquid biopsy are integrated into oncology practice. In AI-enabled precision oncology, key concerns include biased training datasets, lack of transparency, uncertain accountability, and the need for rigorous real-world validation before clinical deployment. In parallel, genomic and liquid-biopsy approaches raise questions regarding data privacy, informed consent, and the management of incidental germline findings that may have implications not only for the patient but also for family members. These issues underscore that innovation in BC care must be accompanied by robust ethical, regulatory, and governance frameworks [241,242,243]. Importantly, these ethical and governance concerns are not peripheral to implementation; they are central determinants of whether emerging technologies can be deployed safely, equitably, and at scale. In this sense, the future direction of BC care is not solely technological, but also organizational, regulatory, and system-dependent [244].

## 7 Conclusion

BC remains a highly heterogeneous and globally consequential disease whose effective management requires integration of molecular biology, diagnostic precision, therapeutic innovation, and long-term survivorship care. Advances in the understanding of tumor subtypes, oncogenic signaling pathways, genomic and epigenetic alterations, and tumor–microenvironment interactions have substantially refined the biological framework through which BC is classified and treated. In parallel, important progress in imaging, pathology, molecular profiling, liquid biopsy, and AI has expanded the possibilities for earlier detection, improved risk stratification, and more individualized treatment planning.

Therapeutically, the field has moved beyond a largely uniform treatment paradigm toward increasingly personalized strategies that combine surgery, radiotherapy, endocrine therapy, chemotherapy, targeted agents, and immunotherapy according to disease subtype and molecular vulnerability. These developments have improved outcomes for many patients and have reinforced the importance of adaptive, multimodal approaches capable of addressing tumor evolution over time. Nevertheless, treatment resistance, intratumoral heterogeneity, and the incomplete translation of emerging biomarkers into routine practice remain major barriers to durable clinical benefit. At the same time, the growing population of BC survivors has highlighted the need to view cancer care beyond disease control alone. Quality of life, long-term toxicities, recurrence risk, psychosocial burden, and equitable access to survivorship services are now central components of comprehensive BC management. Persistent disparities in screening, diagnosis, treatment delivery, and access to innovation continue to shape outcomes across and within countries, underscoring that scientific progress alone is insufficient if not accompanied by effective implementation.

Overall, the future of BC care will depend on the successful integration of precision medicine with robust health systems, evidence-based policy, and equitable access to care. Emerging technologies and biomarker-driven platforms hold substantial promise, but their impact will depend on rigorous validation, standardization, clinical utility, and affordability in diverse settings. Thus, meaningful improvement in BC outcomes will require not only continued scientific discovery, but also coordinated efforts to ensure that innovation is translated into safe, effective, and accessible care for all patients.

## Data Availability

Not applicable.
